# Multi-omics revealed the effects of dietary energy levels on the rumen microbiota and metabolites in yaks under house-feeding conditions

**DOI:** 10.3389/fmicb.2023.1309535

**Published:** 2024-01-09

**Authors:** Xiaoyong Ma, Yongfu La, Guowu Yang, Rongfeng Dai, Juanxiang Zhang, Yonghui Zhang, Jiaming Jin, Xiaoming Ma, Xian Guo, Min Chu, Ping Yan, Qiang Zhang, Chunnian Liang

**Affiliations:** ^1^Key Laboratory of Yak Breeding Engineering Gansu Province, Lanzhou Institute of Husbandry and Pharmaceutical Science, Chinese Academy of Agricultural Sciences, Lanzhou, China; ^2^Key Laboratory of Animal Genetics and Breeding on Tibetan Plateau, Ministry of Agriculture and Rural Affairs, Lanzhou, China; ^3^Gansu Grassland Technical Extension Station, Lanzhou, China; ^4^Institute of Animal Husbandry and Veterinary, Tibet Autonomous Regional Academy of Agricultural Sciences, Lhasa, China

**Keywords:** dietary energy levels, house feeding, rumen microbial, 16S rRNA, metagenome, metabolomics, yak

## Abstract

Yak (*Bos grunniens*) is a unique large ruminant species in the Qinghai-Tibetan Plateau (QTP). Changing the energy levels of their rations can significantly improve their growth performance. Therefore, studying the effects of dietary energy levels on the rumen microflora and metabolites of yak is crucial for enhancing the development of the yak industry. Currently, there is a lack of understanding regarding the impact of feeding energy diets on rumen fermentation parameters, microbial functions, and metabolites. This study was designed to determine the appropriate energy level for feeding yak. Three test diets with metabolizable energy levels of 7.57 MJ/kg, 9.44 MJ/kg, and 11.9 MJ/kg were used and the concentration of volatile fatty acids (VFA) in rumen fluid was measured. The microbial communities, functions, and metabolites in yaks were studied by 16S rRNA sequencing, metagenome, and LC-MS non-targeted metabolomics to investigate the relationships among rumen fermentation parameters, microbial diversity, and metabolites. Ration energy levels significantly affect total VFA, acetate, propionate, butyrate, *iso*-valerate, valerate, and acetate/propionate (*p* < 0.05). At the phylum level, the dominant phyla in all three treatment groups were Bacteroidota, Firmicutes, and Actinobacteriota. At the genus level, the abundance of the *unclassified_o__Bacteroidales*, *norank_f_Muribaculaceae*, *Lachnospiraceae_NK4A136_group*, and *Family _XIII_AD3011_group* showed significant differences (*p* < 0.05) and were significantly correlated with differential metabolites screened for phosphatidylcholine [PC(16:0/0:0), PC(18:3/0:0)], uridine 3′-monophosphate, and adenosine monophosphate, etc. CAZymes family analysis showed that GHs and CEs differed significantly among the three groups. In addition, differential metabolites were mainly enriched in the pathways of lipid metabolism, nucleotide metabolism, and biosynthesis of other secondary metabolites, and the concentrations of differential metabolites were correlated with microbial abundance. In summary, this study analyzed the effects of ration energy levels on rumen microorganisms and metabolites of yaks and their relationships. The results provided a scientific basis for the selection of dietary energy for yaks in the house feeding period in the future.

## 1 Introduction

The yak (*Bos grunniens*) is a unique large ruminant species to the Qinghai-Tibet Plateau (QTP), it was domesticated by the nomads of the QTP about 7,300 years ago. During its long-term evolution, it has adapted to the harsh environment of the QTP, such as low oxygen, high cold, and strong ultraviolet radiation. It is closely related to the food, clothing, housing, and transportation of herdsmen (Xiong et al., 2014; Qiu et al., 2015). The forage grass in alpine meadow areas experiences a withering period that lasts up to 7 months, during this time, the yield and energy level of the forage grass decrease, leading to a decline in the weight of yaks. Some yaks may even die if they are unable to access forage grass for a long time, which causes herdsmen to suffer serious economic losses (Long et al., 1999; Xue et al., 2005). Therefore, exploring the method of house-feeding fattening is crucial for safeguarding the life and property of herdsmen. Maintaining a reasonable dietary energy level is one of the important approaches to enhance the fattening effect of yaks.

The rumen compartment of ruminants contains a large number of microbial communities consisting mainly of bacteria, archaea, ciliated protozoa, and phages (Ley et al., 2008; Mccann et al., 2014). Some of these floras are capable of fermenting and converting indigestible plant polysaccharides into volatile fatty acids (VFAs) and microbial proteins that provide nutrients to the host (Mizrahi and Jami, 2018; Seshadri et al., 2018; Andersen et al., 2020; Yu et al., 2020). The complex rumen microecology is influenced by a variety of factors such as diet, breed, age, and altitude (Lin et al., 2019; Zhang et al., 2019). Among them, the composition of the microbial community is susceptible to the change of ration nutrient composition (Mohammed et al., 2014; Zhang J. et al., 2017). It has been reported that the relative abundance of dominant bacteria in yaks’ rumen changes with the increase in dietary energy level, accompanied by the up-regulation of the rumen epithelial VFA transporter gene (Ahmad et al., 2020). In addition, rumen microbial structure is an important indicator of microbial function and metabolites (Bannink et al., 2016). Previous studies have shown that approximately 60% of the metabolites in rumen fluid are produced by rumen microbes (Saleem et al., 2013). These compounds accurately reflect the relationship between the host, rumen microbes, and dietary levels. However, there are fewer studies on the effect of dietary energy levels on rumen flora function and metabolites in yak. Therefore, the present study aims to address this aspect.

Since the homeostasis of the internal environment of the rumen is critical for healthy animal growth, analysis of rumen microbes and metabolites can help explain the functional properties of microbiota composition and metabolites in the host under specific conditions and will provide important insights for improving feed conversion efficiency in yaks (Heinken et al., 2013; Zhang R. et al., 2017). In recent years, an increasing number of researchers have adopted a combined multi-omics analysis approach to reveal the effect of ingested dietary nutrients on animal growth performance and rumen microbes (Xue et al., 2020). In one study, macrogenomics and metabolomics were used to analyze rumen microbiota in yak and it was found that dietary crude protein levels affect metabolites associated with bile secretion and histidine metabolism in the rumen, and these compounds were associated with rumen dominant microbiota (Dai et al., 2023). Similarly, a multi-omics analysis of rumen fluid from Holstein heifers revealed a reduction in the relative abundance of cellulose-degrading bacteria in the rumen with higher dietary concentration. Additionally, a correlation was observed between the affected microbiota and candidate metabolites Zhang J. et al. (2017).

While microbiome and metabolomics research methods have been extensively employed in ruminants, there is a scarcity of studies investigating the impact of varying dietary energy levels on the composition of rumen microflora and metabolites in yaks. As yaks permanently reside in the plateau throughout the year, the unique growing environment of yaks make it possible that the research results of other bovine species under similar feeding conditions may not apply to yaks. In addition, most of the studies on diet treatment only analyzed the composition of rumen microorganisms, and did not conduct in-depth research on their functions and metabolites. Therefore, in this study, 16S rRNA sequencing technology, metagenomics and LC-MS non-targeted metabolomics data were combined to explore the effects of three dietary energy levels on rumen microflora and function and to analyze the effects of dietary energy levels on growth indexes and rumen fermentation parameters of yaks. The correlation between rumen microflora and differential metabolites was analyzed, and the interaction between microorganisms and metabolites was systematically discussed. It provides more theoretical guidance for yak fattening in the hay period and helps herdsmen to increase economic benefits.

## 2 Materials and methods

The animal experiments involved in this experiment were approved by the Lanzhou Institute of Husbandry and Pharmaceutical Sciences of the Chinese Academy of Agricultural Sciences (CAAS) (approval number: 1610322020018). All sampling procedures are strictly in accordance with the Guidelines for Ethical Treatment of Experimental Animals in China.

### 2.1 Animals, diets, and experimental design

The Liqiaru Livestock Farm in the Tibetan Autonomous Prefecture of Gannan, Gansu Province, China, served as the site of this experiment. We have chosen 30 healthy male yaks who are 6 months old as our study subjects. Prior to the pre-test, the average body weight was 58.15 ± 6.20 kg. Using R software (Version 4.1.2), the 30 yaks were randomly separated into three groups based on body weight. Each group was fed diet with low energy level (LG: Neg 1.77 MJ/kg), medium energy level (MG: Neg 3.88 MJ/kg), and high energy levels (HG: Neg 5.28 MJ/kg), respectively. According to the Chinese Beef Cattle Feeding Standard (NY/T 815-2004), the diet formula was created. In the Table 1, the three diets’ nutritional makeup and composition are displayed. During the experiment, the amount of feed given to the yaks was adjusted by 1.2% based on their monthly body weight data. The trial period was 150 days long, with a 15-day pre-test phase. The body weight of yaks was measured regularly on the day of the end of the pre-test and every month during the trial.

**TABLE 1:** Diet composition and nutrient content.

Raw material name	Dry matter base/%		
	LG	MG	HG
Oat grass	84.16	37.80	14.93
Palm kernel cake	0.00	7.96	11.44
Fat soybean oil	0.00	0.00	1.46
Corn bran	2.01	4.02	0.00
Corn starch 67	1.99	29.84	55.88
Magnesium oxide	0.08	0.11	0.28
Wheat bran	0.00	5.59	0.00
Molasses	0.00	1.02	2.12
Sodium bicarbonate	0.42	0.79	0.00
Calcium carbonate	0.11	0.56	0.68
Salt	0.42	0.67	0.96
Urea CP280	0.56	0.34	0.00
Cottonseed meal CP42	4.58	2.03	0.00
Calcium bicarbonate dicalcium	0.51	1.12	1.69
Mineral element vitamin premix	0.16	0.64	0.38
Soymeal CP44	4.53	3.02	4.03
Barley PI65	0.00	4.48	0.00
Soybeans	0.00	0.00	6.17
**Nutrient composition of DM**			
Dry matter DM (%)	100.00	100.00	100.00
Crude protein CP (%)	11.94	12.08	12.21
Ruminal protein (%)	51.23	52.30	61.84
Soluble protein (%)	37.31	35.48	23.03
Metabolizable energy (MJ/Kg)	7.57	9.44	11.90
Net energy for maintenance (MJ/Kg)	4.88	6.08	7.67
Acid detergent fiber ADF (%)	37.89	24.32	15.44
Neutral detergent fiber NDF (%)	57.00	40.03	25.68
Non-Fiber Carbohydrate NFC	24.76	38.84	49.44

The premix provided the following per kg of the diet: VA 3500 IU, VD 1000 IU, VE 40 IU, Mn 40 mg, Fe 50 mg, Cu 10 mg, Zn 40 mg, Se 0.3 mg, others are estimates.

At the end of the experiment, 6 yaks with similar body weights were selected from each group, and rumen fluid was collected by using a gastric tube sampler. filtered with 4 layers of sterilized gauze, and each yak rumen fluid was divided into 3 tubes of centrifuge tubes (15 mL), labeled, and placed in a liquid nitrogen tank, transported back to the laboratory and stored at −80°C for subsequent sequencing analysis and VFAs determination.

### 2.2 Determination of fermentation parameters of rumen fluid

The rumen fluid was thawed at room temperature and centrifuged (15,000 × *g*, 4°C, 15 min), 1 mL of supernatant was added to a 1.5 mL centrifuge tube, followed by the addition of 0.2 mL of 25% metaphosphoric acid solution containing internal standard (2-Ethylbutyric acid, 2 EB) and centrifugation again (15,000 × *g*, 4°C, 15 min). The concentration of volatile fatty acids (VFAs) was determined using gas chromatography with reference to the method described by Erwin et al. (1961).

### 2.3 DNA extraction of rumen microbial

The rumen fluid samples were thawed at 4°C and the total DNA from the sample genome was extracted following the instructions of the DNA extraction kit Fast (DNA^®^ SPIN Kit for soil, MP Biomedicals, Santa Ana, CA). First, 0.5 g sample, 978 μL sodium phosphate buffer and 122 μL MT buffer were added to the lysis matrix E tube, oscillates in the MP grinder (FastPrep-24 5G, MP, America) for 40 sec at a speed of 6.0; centrifuge at 14,000 rpm for 10 min at room temperature; the supernatant was transferred to a 1.5 mL centrifuge tube, 250 μL PPS was added and mixed; centrifuge again at room temperature at 14,000 rpm for 5 min. The supernatant was transferred to a 2 mL tube containing 900 μL Binding Matrix, mixed, and turned upside down for 3 min; instantaneous centrifugation for 5 s, carefully discard the supernatant; add 500 μL 5.5M guanidine isothiocyanate solution, mix well, transfer to SPINTM Filter; add 500 μL SEWS-M, centrifuge at 14,000 rpm for 1 min, discard the filtrate, and repeat the washing; abandon the liquid in the collection tube, centrifuge at 14,000 rpm for 3 min, remove the residual solution, and dry for 3 min; 100 μL DES eluent preheated at 55°C was added and allowed to stand for 5 min. After centrifugation at 14,000 rpm for 2 min at room temperature, the SPINTM Filter was discarded to obtain total DNA. The purity of the DNA was measured using the NanoDrop2000 (NanoDrop Technologies, Wilmington, DE, USA), while the concentration of the DNA was measured using the TBS-380 microfluorometer (Turner Biosystems, USA). The integrity of the DNA was assessed by 1% agarose gel electrophoresis. The DNA was fragmented into fragments of approximately 400 bp using an ultrasonic disruption device (Covaris M220).

### 2.4 16S rRNA gene sequencing and bioinformatics analysis

PCR amplification was performed using the universal primers 338 F (5-ACTCCTACGGGGAGGCAGCAG-3) and 806 R (5-GGACTACHVGGGTWTCTAAT-3), which targeted the V3-V4 region of the bacterial 16S rRNA gene. The PCR products were purified with the AxyPrep DNA Gel Recovery Kit (Axygen Biosciences, Union City, CA), and initial quantitative analyses were based on electrophoresis results. The PCR products were quantified using the QuantiFluor TM-ST blue fluorescence quantitative system from Promega (United States) and mixed to meet each sample’s sequencing requirements. Library construction employed the TruSeq TM DNA Sample Prep Kit from Illumina (San Diego, CA, United States). We performed sequencing on the Illumina MiSeq PE-250 platform. All the raw data were submitted to the NCBI Sequence Read Archive (SRA) database (Accession number: PRJNA1017979).

The paired end reads (PE reads) obtained from Illumina sequencing underwent splicing via FLASH software^1^ (Reyon et al., 2012). Based on their overlap relationship, the sequences were subjected to quality control and screening using Fastp software^2^ (Chen et al., 2018). The samples were identified based on the barcodes and primers located on both ends of the sequence, and the sequence direction was adjusted accordingly. The Usearch software (version 7.0)^3^ platform was utilized to screen the sequences and conduct Operational taxonomic unit (OTU) statistics (Edgar, 2010). Additionally, the sequences were clustered based on 97% similarity, subsequently, we utilized the RDP Classifier Bayesian algorithm (version 2.11),^4^ in combination with the Silva 16S rRNA databases, to classify and annotate the sequences, and we analyzed the community composition of the samples at each classification level (Wang et al., 2007). Alpha diversity analysis was conducted with Mothur software (1.30.2),^5^ partial Least squares-discriminant Analysis (PLS-DA) was used to distinguish the observed values between groups (Schloss et al., 2009).

### 2.5 Metagenomic sequencing and bioinformatics analysis

The PE library was built following the TruSeq TM DNA Sample Prep Kit (Illumina, San Diego, CA, USA), the bridge PCR was executed through the NovaSeq Reagent Kits, and metagenome sequencing was performed utilizing the Illumina HiSeq Xten sequencing platform. Shanghai Meiji Biomedical Technology Co., Ltd. (Shanghai, China) conducted the library construction, as well as the sequencing for 16S and metagenome purposes.

Fastp software (see text footnote 2, version 0.20.0) was used to cut the adapter sequences at the 3′ and 5′ ends of reads (Chen et al., 2018). After shear, reads with a length of less than 50 bp, an average base mass value of less than 20 and N bases were removed, and high-quality pair-end reads and single-end reads were retained; the reads were compared to the yak genomic DNA sequence,^6^ and contaminated reads with high similarity were removed using BWA^7^ (version 0.7.9a) (Li and Durbin, 2009), the final assembly results were obtained by the MEGAHIT^8^ (version 1.1.2) splicing software, which assembled optimized sequences (Li et al., 2015). Only contigs ≥ 300 bp were selected. The gene sequences expected from all samples (parameters: 90% identity, 90% coverage) were clustered using CD-HIT (Fu et al., 2012). In each class, the longest gene is selected as the representative sequence to construct a non-redundant gene set. The SOAPaligner software was utilized to compare the high-quality reads of each sample to the non-redundant gene set (95% identity), the abundance of each gene in the corresponding sample was then statistics (Li et al., 2008). A total of 801,978,638 original sequences were obtained from the metagenomic sequencing data of 18 samples, each sample had 44,554,369 ± 3,096,896 (mean ± SD) original sequences. After quality control, a total of 633,829,290 optimized sequences were obtained after removing the host genome, with an average of 35,212,738 ± 3,031,722 (mean ± SD) optimized sequences, 79.07% ± 4.63% (mean ± SD) of the original sequence.

### 2.6 Functional annotation

Annotation of carbohydrate-activating enzymes (CAZy)^9^ using Diamond (Version 2.0.13) (comparison parameter set expectation e-value of 1e-5), obtaining the relative abundance of CAZyme.

### 2.7 Determination and analysis of rumen fluid non-target metabolome

First, transfer 200 μL of the sample to a 1.5 mL centrifuge tube, then, add 800 μL of extract (methanol: acetonitrile = 1:1, v/v) and vortex mix for 30 s, extract the sample by low-temperature ultrasonic for 30 min (5°C, 40 KHz); next, precipitate the samples at −20°C for 30 min and centrifuge for 15 min (13,000 *g*, 4°C, Eppendorf, Centrifuge 5430R), the liquid remaining after centrifugation was moved to a new centrifuge tube and dried using nitrogen purging (JXDC-20, Shanghai, China), the dried sample was then reconstituted in 120 μL of reconstitution solution (acetonitrile: water = 1:1), the sample was mixed using vortexing for 30 s and low-temperature ultrasonic extraction for 5 min at 5°C, 40 KHz. The sample was centrifuged for 10 min at 13,000 *g*, 4°C prior, The supernatant was transferred to the injection vial with intubated for analysis. The instrument platform used for LC-MS non-targeted metabolomics analysis was an ultra-high performance liquid chromatography tandem Fourier transform mass spectrometry system (UHPLC, Q-Exactive HF-X, ThermoFisher Scientific, Waltham, MA, USA). A total of 3 μL sample was separated by HSS T3 column (100 mm × 2.1 mm i.d., 1.8 μm) and detected by mass spectrometry, the mobile phase A was 95% water + 5% acetonitrile (containing 0.1% formic acid), and the mobile phase B was 47.5% acetonitrile + 47.5% isopropanol + 5% water (containing 0.1% formic acid). Sample mass spectrometry signal acquisition was performed in positive (ESI +) and negative (ESI-) ion scanning modes, with a mass scan range of 70–1,050 m/z. 50 psi, the auxiliary gas flow rate was 13 psi, the auxiliary gas heating temperature was 425°C, the positive-mode ion spray voltage was set at 3,500 V, the negative-mode ion spray voltage was set at −3,500 V, the ion transfer tube temperature was 325°C, and the normalized collision energy was 20-40-60 V cyclic collision energy. The resolution of the primary mass spectrum was 60,000 and the resolution of the secondary mass spectrum was 7,500, and the data were collected in DDA mode. Additionally, 20 μL of supernatant was taken from each sample and combined as a quality control sample. The raw data were imported into the metabonomics processing software ProgenesisQI (Waters Corporation, Milford, USA) for processing, and finally, a data matrix containing retention time, mass-to-charge ratio, and peak intensity was obtained. Eigenvalues with missing values > 20% in each group were eliminated and missing values were filled with minimum values, the data was normalized using the sum method, and a QC verification RSD ≤ 30% was retained, subsequently, statistical analysis was conducted after the log10 transformation, Substitution tests were performed using Partial Least Squares Discriminant Analysis (PLS-DA). The Kyoto Encyclopedia of Genes and Genomes (KEGG)^10^ database was utilized to confirm the identification of differentially expressed metabolites and assess the impacts of varying energy levels on metabolites.

### 2.8 Relevance analysis

Differential metabolites of VIP > 2, *p*-value < 0.05 were selected and based on Bray_Curtis distance, spearman correlation analysis was performed with dominant microbial, differential microbial genus and rumen fermentation parameters (TVFA, acetic acid, propionic acid, *iso*-butyric acid, butyric acid, isovaleric acid, valeric acid, Acetate/Propionate) and the correlation was visualized by clustering heat map.

### 2.9 Statistical analysis

Yak body weight data were processed by EXCEL, and the average daily gain of each yak was calculated. Taking the group as the independent variable, the monthly body weight and the average daily gain as the dependent variable, SAS 9.2 software was used for one-way analysis of variance, resulting in means ± SEM error values. Groups were used as independent variables, and VFAs data were used as dependent variables, the VFAs were subjected to one-way ANOVA using SPSS software (Version 26) to determine group differences, with means ± SEM error values reported. Statistical significance was indicated by *p* < 0.05.

## 3 Results

### 3.1 Growth index analysis

Body weight and average daily gain of yaks in three energy groups are included in Table 2. Results indicate that the body weight gain of yaks in HG group was significantly higher than that in LG group during the experimental phase (*P* < 0.05), while the difference between HG and MG group is insignificant (*P* > 0.05); a comparison of the Average daily gain shows that yaks in the HG group have statistically significantly higher daily weight gain than those in the LG and MG groups (*P* < 0.05).

**TABLE 2:** Comparison of changes in body weight and average daily weight gain of yaks in three treatment group groups.

Item	Day of experiment	Groups			*p*-Value
		LG	MG	HG	
Body weight	0 d (kg)	58.92 ± 3.89	64.48 ± 4.66	61.94 ± 5.28	0.705
	60 d (kg)	65.10 ± 4.79^b^	74.40 ± 6.53^ab^	85.70 ± 6.37^a^	0.031
	90 d (kg)	73.64 ± 6.23^b^	82.12 ± 6.82^ab^	100.48 ± 7.65^a^	0.018
	120 d (kg)	77.62 ± 6.83^b^	89.82 ± 7.90^ab^	109.52 ± 9.48^a^	0.017
	150 d (kg)	96.08 ± 8.07^b^	104.36 ± 8.90^ab^	127.28 ± 11.14^a^	0.038
Average daily gain (kg/d)		0.25 ± 0.03^b^	0.27 ± 0.03^b^	0.43 ± 0.07^a^	0.048

Values for each treatment group are expressed as mean ± standard error (SEM). In the same row, superscripts with different lowercase letters indicate significant differences between groups.

### 3.2 Changes in rumen fermentation parameters

The concentrations of acetate, propionate, *iso*-valerate and valerate were significantly higher in the HG group than in the other two groups, and the concentration of total VFA and butyrate was significantly higher than in the LG group (*P* < 0.05); acetate/propionate was significantly higher in the LG group than in the MG group, which was significantly higher than in the HG group (*P* < 0.05), and the content of *iso*-butyrate was not statistically significant among the three treatment groups (Table 3).

**TABLE 3:** Effect on rumen fermentation parameters in yak of diet with different energy content.

Item	Treatment group (Mean ± SEM)			*p*-Value
	LG	MG	HG	
Total VFA (mM)	53.55 ± 0.69^b^	52.81 ± 3.44^ab^	75.96 ± 2.96^a^	0.001
Acetate (mM)	40.38 ± 0.31^b^	36.17 ± 3.36^b^	50.79 ± 1.55^a^	0.008
Propionate (mM)	6.96 ± 0.27^b^	8.32 ± 0.87^b^	13.97 ± 0.94^a^	0.001
*Iso*-butyrate (mM)	0.62 ± 0.01	0.72 ± 0.09	0.84 ± 0.05	0.131
Butyrate (mM)	4.54 ± 0.35^b^	6.32 ± 0.81^ab^	8.13 ± 0.58^a^	0.017
*Iso*-valerate (mM)	0.71 ± 0.03^b^	0.73 ± 0.03^b^	1.23 ± 0.09^a^	0.001
Valerate (mM)	0.35 ± 0.02^b^	0.56 ± 0.10^b^	1.00 ± 0.08^a^	0.002
Acetate/Propionate	5.82 ± 0.18^a^	4.37 ± 0.26^b^	3.65 ± 0.16^c^	0.001

The values of each treatment group were expressed as mean ± standard error (SEM). In the same line, the superscripts of different lowercase letters showed significant differences between groups.

### 3.3 16S rRNA sequencing analysis

**FIGURE 1:**
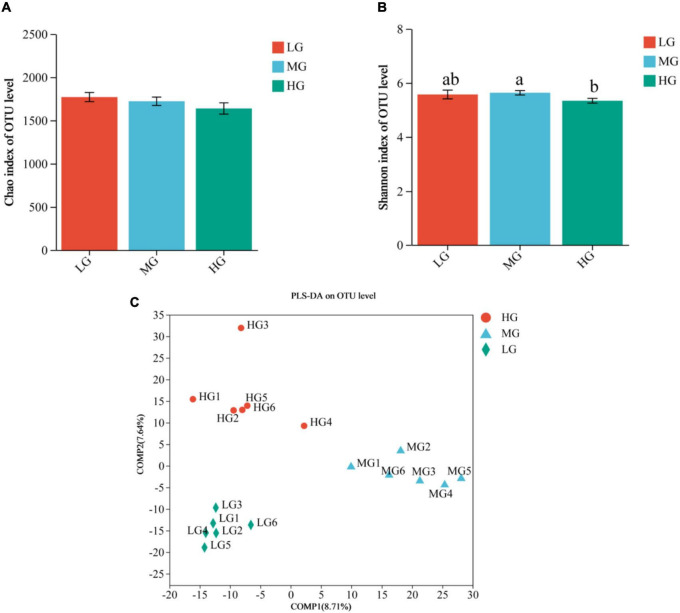
Rumen microbial communities of the three treatment groups were distinguished based on Alpha diversity and PLS-DA analysis. Differences in Chao1 **(A)** and Shannon **(B)** indices of microorganisms in the three treatment groups, different lowercase letters indicate significant differences (*P* < 0.05). **(C)** PLS-DA score plot showing good separation between groups.

The sequencing data of 18 samples were splicing, filtering, and chimera discarding and 949,241 optimized sequences were obtained, with an average length of 416 bp. The representative sequences of OTUs were classified and analyzed at the 97% similarity level using the RDP classifier Bayesian algorithm, resulting in the acquisition of 2,926 OTUs. Alpha diversity analysis indicated variations in microbial diversity and abundance among the three treatment groups, the Chao1 index showed that the LG group had a higher microbial abundance compared to the other two groups, but the difference was not statistically significant. On the other hand, the Shannon’s index indicated that the MG group had significantly higher microbial diversity than the HG group (*P* < 0.05) (Figures 1A, B). The results of the Partial Least Squares Discriminant Analysis (PLS-DA) indicate significant variations in the composition of rumen flora among the three dietary groups in yaks. Furthermore, it is noteworthy that the MG group exhibited more pronounced differences compared to the other two groups (Figure 1C). The analysis revealed distinct disparities and formed well-defined clusters.

### 3.4 Microbial composition of the three treatment groups

**FIGURE 2:**
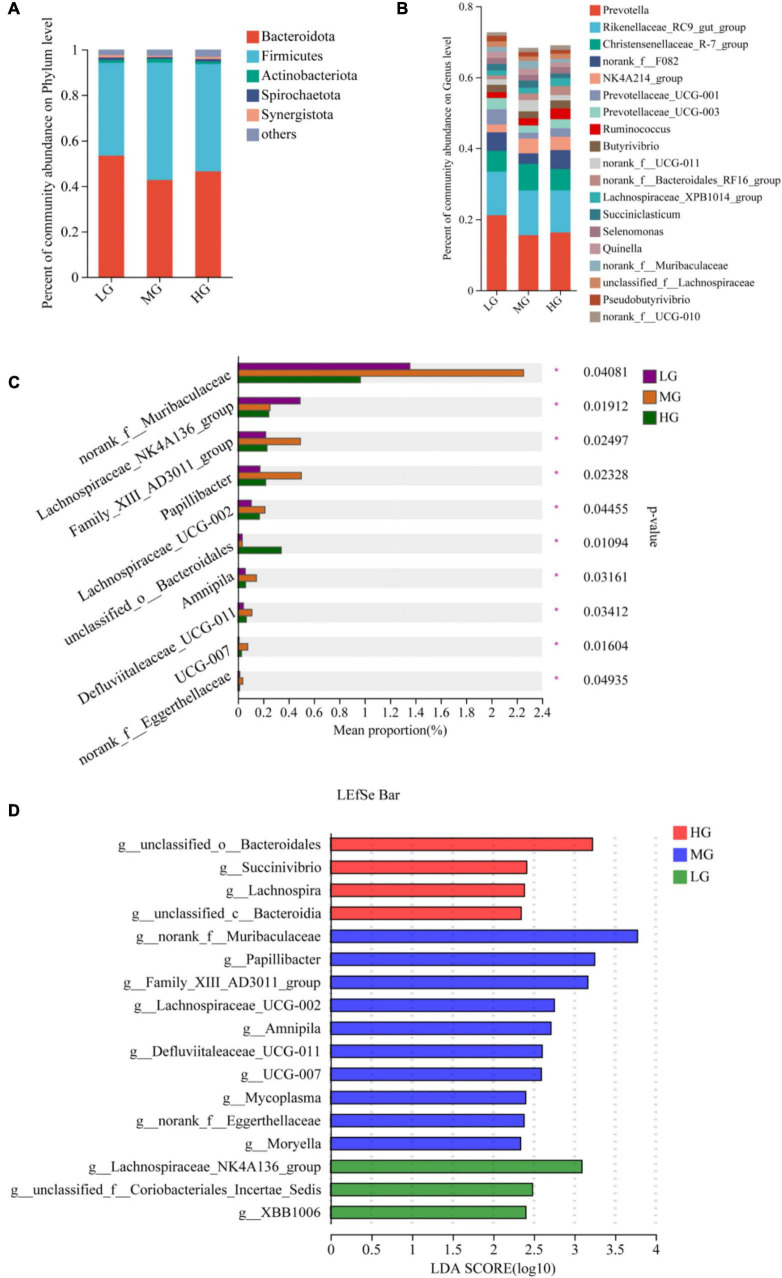
Microbial community composition and differential analysis of the three treatment groups. Percentage of microbial community abundance at phylum **(A)** and genus **(B)** levels for the three treatment groups. **(C)** Kruskal-Wallis H test to compare differences in microbial composition of the three treatment groups at the genus level. **(D)** The most representative biomarkers in each treatment group were identified at the genus level using the LDA Effect Size (LEfSe) algorithm. *Indicates that Microbials are significantly different among groups.

A total of 22 phyla, 163 families, and 342 genus of microbiota were identified in the three treatment groups. Venn diagrams were employed to visually represent the shared and unique number of phylum and genus levels among the three groups, the statistical analysis was conducted using R language tools (version 3.3.1). At the phylum level, there were 20 floras in all three treatment groups, with one colony being identical in both the HG and MG groups. Notably, the p__Cloacimonadota colony was exclusively found in the HG group (Supplementary Figure 1A). At the genus level, the HG group displayed the highest number of exclusive floras with 21, while the MG and LG groups had 10 and 3 exclusive floras, respectively (Supplementary Figure 1B). At the phylum level, the most abundant flora (with a relative abundance greater than 1%) in the three treatment groups were Bacteroidota, Firmicutes, and Actinobacteriota (Figure 2A). Similarly, at the genus level, the dominant flora consisted mainly of *Prevotella*, *Rikenellaceae_RC9_gut_group*, *Christensenellaceae_R-7_group*, *norank_f__F082*, *NK4A214_group*, *Prevotellaceae_ UCG-001*, *Prevotellaceae_UCG-003*, and *Ruminococcus*. The microbial community’s composition in the three treatment groups is shown in Figure 2B. The bar charts indicate that the three groups have similar dominant genus but differ in their relative abundance. The Kruskal-Wallis rank sum test, conducted on microbial genera samples from the three treatment groups (Figure 2C), revealed that *unclassified__o_Bacteroidales* exhibited significantly higher levels in the HG group than in the LG and MG groups (*P* < 0.05). In the MG group, *norank_f__Muribaculaceae*, *Family_XIII_AD3011_group*, *Papillibacter*, *Lachnospiraceae_UCG-002*, *Amnipila*, and *Defluviitaleaceae_UCG-011*, the levels of 6 bacterial groups were significantly higher than those of the other two groups (*P* < 0.05). Similarly, the *Lachnospiraceae_NK4A136_group* in the LG group manifested significantly higher levels than the other two groups (*P* < 0.05). A total of 24 microorganisms exhibiting significant differences within the three treatment groups underwent screening using Linear Discriminant Analysis Effect Size (LEfSe, with LDA threshold of 2). The differential microorganisms uncovered in the LEfSe analysis encompassed the flora observed in the rank-sum test, affirming the differential microorganisms in the various energy groups (Figure 2D).

### 3.5 CAZyme composition

**FIGURE 3:**
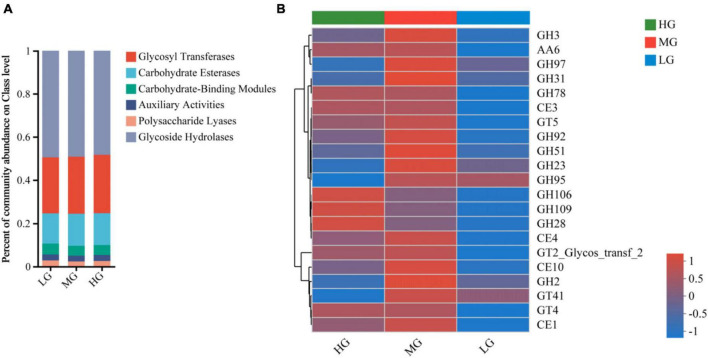
Metagenomic data from the three treatment groups were subjected to carbohydrate active enzyme analysis. **(A)** The proportion of CAZyme abundance at the class level in the three treatment groups. **(B)** The proportion of CAZyme abundance at family level in the three treatment groups.

Ruminal microorganisms can produce various complex CAZymes, accessing information on CAZymes gene annotation is critical to uncovering the mechanism behind microbial carbohydrate metabolism. A total of 524 CAZyme genes were identified in this study. There were 258 Glycoside Hydrolases (GHs), 89 Glycosyl Transferases (GTs), 79 Polysaccharide Lyases (PLs), 66 Carbohydrate Binding Modules (CBMs), 15 Carbohydrate Esterases (CEs) and 19 Auxiliary Activities (AAs). Figure 3 illustrates alterations in the relative abundance of CAZymes responsible for breaking down diets with varying energy levels within different classes and families. At the class level, the LG group had a higher ratio of PLs and CBMs, while the MG group had significantly increased functional abundance of GHs, CEs, GTs, and AAs. In comparison, only AAs and GTs were abundant in the HG group (Figure 3A). Figure 3 illustrates the changes in the relative abundance of carbohydrate enzymes responsible for the decomposition of diet with different energy levels in different classes and families. At the class level, the proportion of GHs, CBMs and PLs in the LG group was higher, while the functional abundance of AAs and GTs in the HG group was significantly increased. Only CEs were abundant in MG group (Figure 3A).

CAZyme families with abundance greater than 1% were visualized through a Heatmap plot (Figure 3B), revealing significant differences among treatment groups with varying dietary energy levels. GH106, GH109, GH28, and GH78 were highly abundant in the HG group. The abundance of 17 CAZyme families, including GH3, GH97, GH31, CE1, and CE4, increased significantly in MG group. The LG group had lower abundance of 21 CAZyme families, suggesting that unbalanced dietary energy levels can affect genes that encode carbohydrate-active enzymes.

### 3.6 Metabolomics of rumen fluid

**FIGURE 4:**
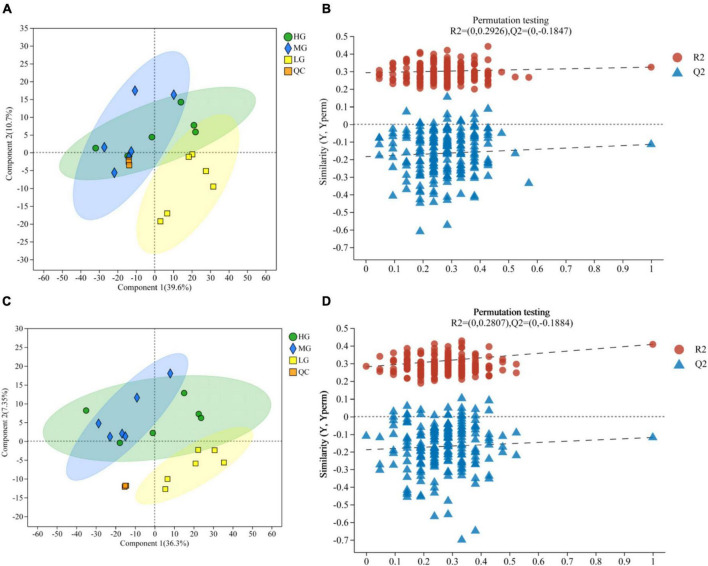
Rumen metabolomics PLS-DA score plots for the three treatment groups (with QC samples). PLS-DA **(A)** score plot and PLS-DA permutation test in cationic mode **(B)**. PLS-DA **(C)** score plot and PLS-DA permutation test in anionic mode **(D)**.

A total of 1,635 metabolites were identified through LC-MS metabolomics analysis. The PLS-DA validated model underwent 200 permutation tests, which were based on PLS-DA scoring of metabolites present in the rumen of the three treatment groups in both cationic and anionic modes (Figures 4A, C): The results indicate proximity between the HG and MG groups, but a clear separation from the LG group. Thus, insights regarding group relationships can be gleaned from the visual examination of a simple fractional spatial clustering model. The intercept of the regression line for Q2 in the PLS-DA validation model is lower than 0, which suggests that the model is reliable and not overfit (Figures 4B, D). Additionally, all of the samples displayed in the PLS-DA score plots lie within the sample confidence circles, indicating that the PLS-DA validation model has high validity.

**FIGURE 5:**
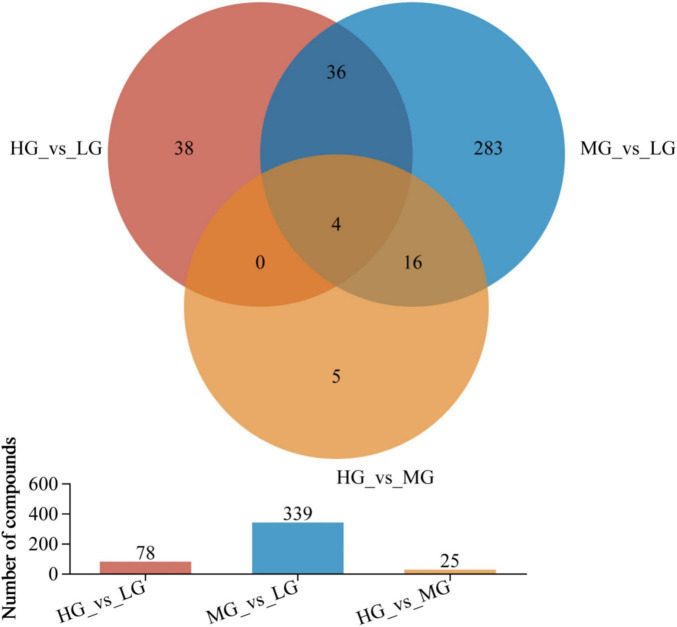
Venn analyzed the compositional characteristics of metabolites in the three treatment groups. In the figure, the different colors represent the differential metabolites in different comparison groups, the overlapping part represents the number of metabolites common to multiple metabolic sets, and the bar graph represents the number of metabolites included in each metabolic set.

In Figure 5, the rumen metabolic profiles of yaks in the MG and LG groups differed significantly, a total of 339 metabolites (210 cations and 129 anions) were detected in the MG and LG groups. This indicates that the amounts of different metabolites varied with the energy level of the diet. A total of 78 species (including 49 cations and 13 anions) were discovered in the comparison of HG and LG, while 25 species (including 12 cations and 13 anions) were identified in the comparison of HG and MG (VIP > 1 and *P* < 0.05), 56 out of 442 differentiated metabolites were found to be shared among the treatment groups, suggesting the influence of dietary levels on rumen metabolites. Supplementary Material 1 (HMDB 4.0)^11^ provided a compound classification of differential metabolites.

**FIGURE 6:**
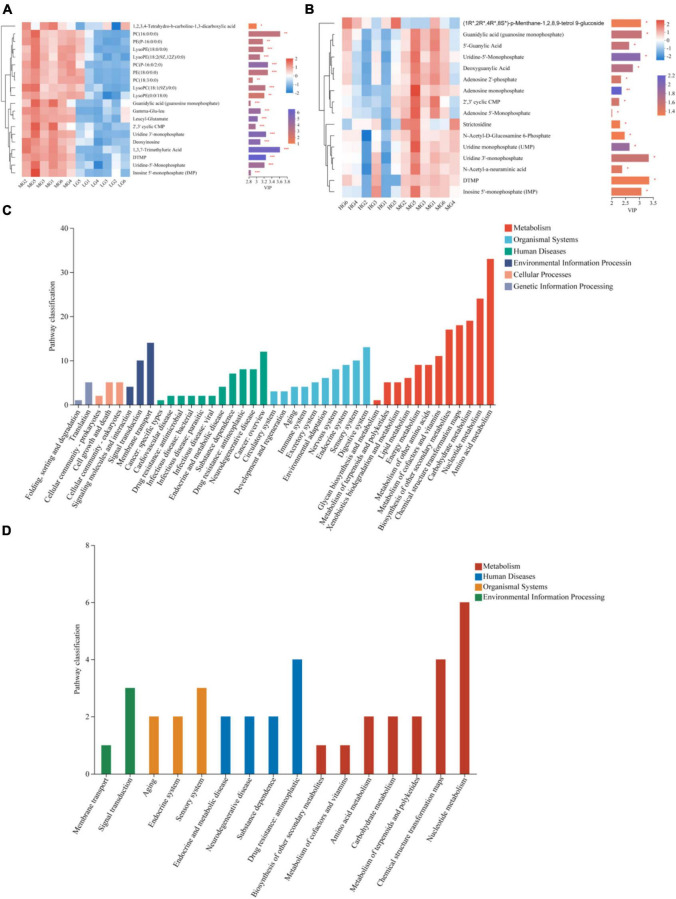
Differential metabolites and metabolic pathways in rumen fluid of three treatment groups. The differences in rumen fluid metabolites between MG vs LG **(A)** and HG vs MG **(B)** are shown by heat maps and VIP values, The tree diagram of metabolites cluster on the left side shows that each column shows a sample, the lower side shows the name of the sample, and each row shows a metabolite. Bar chart showing VIP value on the right. The bars show the different metabolic pathways of the differential metabolites in the rumen fluid of MG vs LG **(C)** and HG vs MG **(D)**. *Indicates *p* < 0.05, **indicates *p* < 0.01, ***indicates *p* < 0.001.

The OPLS-DA model was used to screen the top 20 differential metabolites in the abundance of WG vs LG and HG vs WG, metabolites that have Variable Importance in Projection (VIP) scores greater than 2 were identified as biomarkers. The following metabolites were found in WG vs LG: 1,3,7-Trimethyluric Acid, 5-Thymidylic acid (DTMP), and phosphatidylcholine [PC(16:0/0:0), PC(P-16:0/2:0), PC(18:3/0:0)], as well as Glycerophosphoethanolamines [PE(18:0/0:0) and PE(P-16:0/0)] were also present. Additionally, Uridine 3′-monophosphate and Uridine-5′-Monophosphate metabolites showed significant differences (Figure 6A), and were mainly concentrated in pathways related to Lipid metabolism, Nucleotide metabolism, and Biosynthesis of other secondary metabolites (Figure 6C). Differences in metabolites, such as (1R*,2R*,4R*,8S*)-p-Menthane-1,2,8,9-tetrol 9-glucoside, Uridine 3′-monophosphate, Uridine-5′-Monophosphate, and Guanidylic acid (guanosine monophosphate), were observed between HG and WG groups (Figure 6B). KEGG functional pathway annotation demonstrated significant enrichment in pathways related to Nucleotide metabolism (Figure 6D).

### 3.7 Correlation analysis of microorganisms and rumen fermentation parameters with metabolites

**FIGURE 7:**
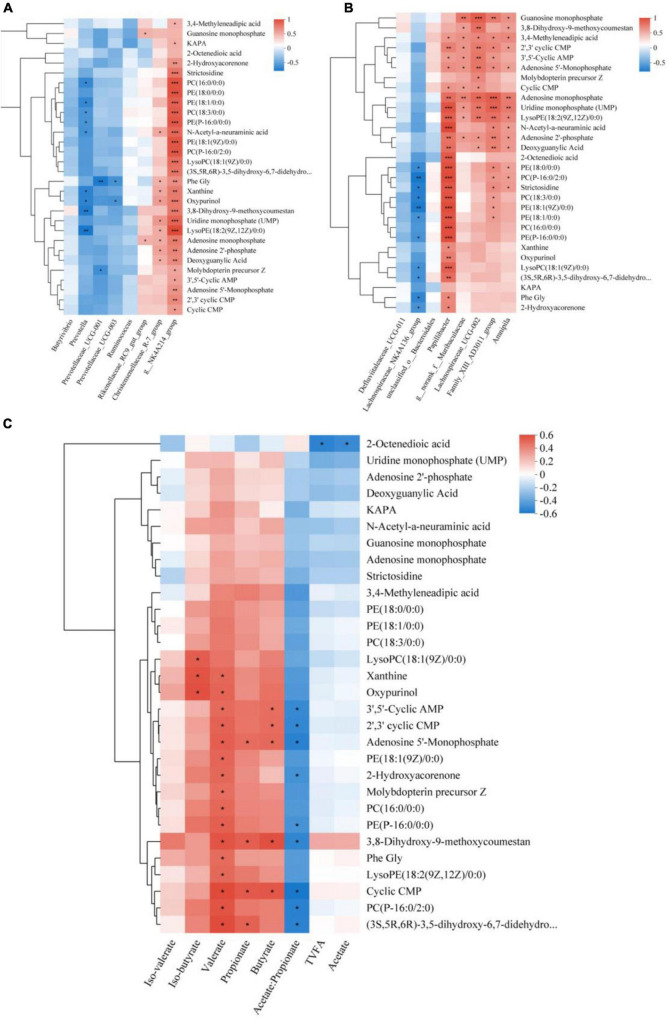
Spearman relationships among rumen microbes, VFAs, and differential metabolites. Spearman correlation between the differential metabolites selected out the three treatment groups and the dominant Microbial **(A)**, differential Microbial **(B)**, and VFAs **(C)**. *Denotes *p* < 0.05, **denotes *p* < 0.01, and ***denotes *p* < 0.001.

Research has revealed that approximately 60% of the metabolites present in rumen fluid are generated by microorganisms inhabiting the rumen (Saleem et al., 2013). Therefore, it is crucial to comprehend the relationship between the two. First, a correlation heat map was constructed using Spearman correlation coefficients to analyze the relationship between the dominant flora and differential metabolites (Figure 7A). The study revealed a significant positive correlation between *g__NK4A214_group* and the top 30 differentially abundant metabolites, the *Christensenellaceae_R-7_group* demonstrated a positive correlation with Uridine monophosphate (UMP), LysoPE (18: 2(9Z,12Z)/0:0), Adenosine monophosphate, Adenosine 2’-phosphate, Deoxyguanylic Acid, Phe Gly, Xanthine, Oxypurinol, and N-Acetyl-a-neuraminic acid. The *Rikenellaceae_RC9_gut_group* exhibited a substantial and positive association with Adenosine monophosphate and Guanosine monophosphate. Additionally, *Prevotella*, *Prevotellaceae_UCG-001*, and *Prevotellaceae_UCG-003* were associated with Molybdopterin precursor Z, LysoPE (18:2(9Z,12Z)/0:0), Oxypurinol, phosphatidylcholine [PC(16:0/0:0), PC(18:3/0:0)], and glycerophosphoethanolamine [PE(18:0/0:0), PE(P-16:0/0:0), PE(18:1/0:0)] metabolites showed significant negative correlations. The differential flora-metabolite associations revealed that *Papillibacter*, *Lachnospiraceae_UCG-002*, *Family_XIII_AD3011_group* and *Amnipila* were positively correlated with the top 30 differential metabolites in abundance, *g___norank_f___Muribaculaceae* showed significant positive correlation with metabolites such as adenosine monophosphate, uridine monophosphate (UMP), LysoPE (18:2(9Z,12Z)/0:0), and N-acetyl-a-neuraminic acid (Figure 7B).

We also conducted a correlation analysis between differential metabolites and rumen fermentation parameters (Figure 7C). Our results indicate that TVFA and Acetate had a negative correlation with metabolites including Uridine monophosphate (UMP), Adenosine 2′-phosphate, and Deoxyguanylic Acid, Propionate was found to be significantly and positively correlated with 3,8-Dihydroxy -9-methoxycoumestan and Adenosine 5′-Monophosphate, as well as with the remaining compounds, Iso-butyrate was significantly and positively correlated with LysoPC (18:1(9Z)/0: 0), Xanthine, and Oxypurinol, Butyrate was also significantly and positively correlated with 3,8-Dihydroxy-9-methoxycoumestan, 3′,5′-Cyclic AMP, 2′,3′ cyclic CMP, and Adenosine 5′-Monophosphate. Iso-valerate was positively correlated with 3,8-Dihydroxy-9-methoxycoumestan, Oxypurinol, and Phe Gly. Additionally, Valerate was significantly and positively correlated with 17 compounds. Negatively correlated metabolites included g. glycerophosphoethanolamine [PE(P-16:0/0:0), PE(18:1/0:0)], 2-Hydroxyacorenone, and others. Acetate/Propionate displayed a negative correlation with these metabolites.

## 4 Discussion

During the dry grass stage on the Tibetan Plateau, the growth performance of yaks decreased under natural grazing conditions, which hindered the development of yak industry. Consequently, there is a pressing need for a change in feeding patterns to promote animal husbandry in the QTP (Long et al., 1999; Xue et al., 2005). Prior research indicates that pre-partum heifers body weight and performance can be enhanced by varying their dietary energy levels (Chen et al., 2022). The study showed that yaks in the MG group had faster monthly growth compared to the LG group, as energy levels increased, yaks in the HG group gained more weight, with a higher average daily gain than the other two groups. This suggests that the energy gradient diets under housed feeding will improve yaks’ growth performance to varying degrees, thus, the excellent traits of yaks will be utilized and the economic benefits of herders will be improved.

Volatile fatty acids (VFA) are products of rumen microbial degradation of substances such as cellulose, pentosans, and proteins, and can provide 70–80% of energy for ruminants (Bainbridge et al., 2018). Diet type and nutritional level, among others, affect the production of VFA (Rabelo et al., 2016). With the increase of the crude fiber content in the diet, the concentration of acetic acid also increased, while the concentrations of propionic acid and butyric acid showed a strong correlation with non-fibrous carbohydrates (NFC) in the diet (Kaufmann and Rohr, 1966). Acetic acid serves as a precursor to fat synthesis in ruminants, whereas propionic acid is crucial for glycogen synthesis in the liver (Young, 1977; Allen, 2014; Liu et al., 2016). An elevated concentration of propionic acid in the rumen is indicative of improved efficiency in energy conversion which is essential for animal weight gain, according to Cui et al. (2023). In this study, the LG group had a lower concentration of propionic acid, likely due to its low non-starch carbohydrate (NFC) content and high neutral detergent fiber (NDF) content. Meanwhile, the HG group exhibited a higher concentration of propionic acid than the other groups, indicating efficient conversion of high-energy diet and accelerated body weight gain. High levels of acetate, propionate, and valerate concentrations have been observed in the rumen of dairy cattle that have high yielding capacity (Xue et al., 2019). Previous research has reported that the concentrations of total VFA and acetate in the rumen of animals fed with high-quality feed increases variedly (Guan et al., 2008). Thus, the elevated level of acetate in the HG group could be attributed to an enhanced growth performance. Additionally, a reduction in ration concentration leads to an increase in the Acetate/Propionate ratio in the rumen (Pang et al., 2022). The LG group exhibited the highest ratio of Acetate/Propionate, aligning with previous findings. Comparatively, Acetate/Propionate in the MG group was lower than the LG, yet higher than the HG, which could have contributed to the suboptimal growth performance of the MG group.

The rumen microbiota plays a critical role in maintaining ruminant health and normal digestive function (Ley et al., 2008). Bacteria in the rumen, the most prevalent and diverse microorganisms, assist fermentation and produce nutrients that provide energy for the host (Saleem et al., 2013; Deusch et al., 2017). This study aims to investigate the impact of feed energy level on yak rumen flora through 16S rRNA high throughput sequencing. Prior studies have reported significant variations in the microbial composition of the host’s gastrointestinal tract in association with different food sources and geographic settings (Amato et al., 2015). Petri et al. (2013) revealed that the rumen core flora in ruminants includes ten bacterial taxa, but their relative abundance may vary. In this study, venn’s analysis showed 20 floras were shared at the phylum level in the three treatment groups, indicating that the core microflora remained unchanged. The diversity index (Shannon) and richness index (Chao1) reflect the distribution of microbial communities in the rumen, and Sonnenburg and Bäckhed (2016) reported that high-fiber rations consumed by ruminants increase microbial diversity in the rumen. We found that the HG group had lower Shannon and Chao1 values compared to the other two groups, which is consistent with previous research. Additionally, PLS-DA analysis revealed that the energy level of the ration played a significant role in the composition of rumen flora, at the phylum level, this study found that Bacteroidota, Firmicutes, and Actinobacteriota were the dominant phyla, which have been reported to make up more than 90% of the bacteria in the gastrointestinal tract (Eckburg et al., 2005). These results are consistent with previous research (Chen et al., 2015; Zhou et al., 2017). Therefore, the bacterial flora operating in the rumen is relatively stable in terms of phylum taxonomy. Bacteroidota express numerous genes that encode carbohydrate-active enzymes, which carry out the fermentation of dietary carbohydrates and produce short-chain fatty acids (SCFA) (Hess et al., 2011; Thomas et al., 2011). In the rumen, Firmicutes is the dominant core bacterium that joins forces with Bacteroidota to break down cellulose and protein (Huo et al., 2014). The abundance of Bacteroidota and Firmicutes in the HG group was lower compared to the other two groups, suggesting a downward trend of both flora with a rise in ration concentration. This aligns with the findings of Nagaraja and Titgemeyer (2007). Actinobacteria has a low abundance within the rumen, however, it plays a crucial role in the formation of biofilms, as well as in the fermentation and digestion of soluble carbohydrates (Pitta et al., 2016).

At the genus level, *Prevotella*, *Rikenellaceae_RC9_gut_group*, *Christensenellaceae_R-7_group*, *NK4A214_group*, *Prevotellaceae_UCG-001*, *Prevotellaceae_UCG-003*, and *Ruminococcus* were the dominant flora among the three treatment groups. *Prevotella* has an important role in protein and starch metabolism and has the ability to utilize hemicellulose (Bekele et al., 2010). Through the results of this study, the abundance of *Prevotella* was increased by the medium energy level diet. Fan et al. (2017) found that *Rikenellaceae_RC9_gut_group* has an important role in protein degradation. In addition, it has been shown that the relative abundance of *Rikenellaceae_RC9_gut_group* increases with increasing diet concentration (Pang et al., 2022). In this experiment, *Rikenellaceae_RC9_gut_group* had the highest abundance in the MG group. Our hypothesis is that the rumen internal environment that develops after feeding yaks with medium-energy level diets is the point of inflection for the linear increase in the abundance of *Rikenellaceae_RC9_gut_group*. According to Liu et al. (2019), *Prevotellaceae_UCG-001* and *Prevotellaceae_UCG-003* were found to have a negative correlation with valeric acid and isovaleric acid and facilitated the resynthesis of branched-chain fatty acids through the elongation of valeric acid and propionic acid or through the alteration of α-ketoacids (Bainbridge et al., 2018). The Valeric acid and isovaleric acid concentration of the LG group was low due to the low feed efficiency of the low-energy diet and possibly due to the degradation of VFA by both floras. Additionally observed that *Prevotellaceae_UCG-001* and *Prevotellaceae_UCG-003* exhibited sensitivity to rumen pH (Mao et al., 2016). Specifically, the high concentration of diet in the HG group led to a decrease in rumen pH, ultimately resulting in a reduction of these two bacterial populations. *NK4A214_group*, a genus under Firmicutes, is an uncharacterized taxon, with no studies on its function (Amat et al., 2021). However, our present study’s correlation analysis revealed that this flora and 30 differential metabolites, including Adenosine monophosphate, Uridine monophosphate (UMP), and LysoPE (18:2(9Z,12Z)/0:0), were significantly and positively associated, these differential metabolites are enriched in pathways such as energy metabolism, nucleotide metabolism, and the biosynthesis of secondary metabolites. We therefore hypothesize that the NK4A214_group plays an important role in energy metabolism and biosynthesis. A study conducted by Biddle et al. (2013) has demonstrated that *Ruminococcus* possesses activities that break down cellulose and hemicellulose, resulting in the production of acetic acid, butyric acid, formate, and hydrogen. Liu et al. (2015) discovered that the abundance of *Ruminococcus* was positively associated with the expression of toll-like receptor (TLR) genes, which stimulate the immune response and sustain the internal environment of the host rumen. Thus, it is possible that *Ruminococcus* played a regulatory role in the HG group in maintaining stability of the rumen endo-environment in yaks as the energy level of the ration increased. During the variance analysis, it was determined that feeding rations with high energy levels notably boosted the abundances of *unclassified_o_Bacteroidales*, this outcome could potentially be linked to the rise in VFA concentration and weight gain in the HG group of yaks. Because *unclassified Bacteroidales* are capable of hydrolyzing starch, degrading proteins, and producing volatile fatty acids (Biddle et al., 2013). The abundance of *Norank_f_Muribaculaceae* was significantly higher in the MG group than in the other two groups, and it belongs to the S24-7 family, which is associated with the degradation of a variety of complex carbohydrates (Lagkouvardos et al., 2019).

Ruminal microbes efficiently break down plant polysaccharides due to their ability to produce CAZymes (Garron and Henrissat, 2019). To identify CAZymes in the rumen, Metagenomic was used for annotation, among them, GHs, GTs, CEs, and CBMs have been identified as the main carbohydrate-hydrolyzing CAZymes in the rumen (Rabee et al., 2020). In this research, GHs, GTs, and CEs were found to be the most prevalent in the three treatment groups. The GHs family can break down glycosidic bonds of carbohydrates through a direct process (Stewart et al., 2018; Khatoon et al., 2022). GTs primarily catalyze activated sugar molecules and specific acceptor molecules to form glycosidic bonds (Andrade et al., 2017; Tomazetto et al., 2020). CBMs, which are molecules that bind to lignocellulose, possess the ability to promote the breakdown of cellulose/hemicellulose polymers by glycoside hydrolases (Khatoon et al., 2022). CEs play a significant role in breaking down sugar side chains and promoting glycoside hydrolase degradation (Biely, 2012). PLs can contribute to the breakdown of carbohydrates by removing polysaccharide esters with the help of CEs, as noted by Tomazetto et al. (2020). The investigation of the CAZymes family indicated notable distinctions among the three treatment groups as the diet’s energy level increased. Overall, the MG group contained more families of GHs, including beta-galactosidase (GH2), alpha-L-fucosidase (GH95), and beta-glucosidase (GH3, GH97, and GH51) for converting cellobiose, as well as enzymes for UDP-GlcNA (GT41), acetyl xylan esterase (CE1), and arylesterase (CE10). Yaks in the MG group may have a greater ability to break down cellulose and hemicellulose, as beta-glucosidase not only hydrolyzes lactose but also plant polysaccharides catalyzed by microbial cellulases (Lee et al., 2012). Previous studies have shown the importance of β-glucosidases, specifically GH3, GH51, and GH97, in converting cellobiose to enhance cellulose hydrolases (Chen H. L. et al., 2012). Acetyl xylan esterase (CE1) is an enzyme that is vital for breaking down hemicellulose. Xylan is a significant constituent of plant hemicellulose, and it is made up of a backbone of β-1,4-linked xylopyranoside residues that are decorated with acetyl side groups (Yoshida et al., 2010). It has been observed that the abundance of CAZymes decreased in the HG group as compared to the WG. Previous research has indicated that feeding high levels of diets may lead to a decrease in CAZymes that hydrolyze specific glycosidic bonds (Barrett et al., 2022; Liang et al., 2023). A decline in the CAZyme family implies a reduction in the hydrolytic ability of rumen microorganisms to break down diets. The primary colonies in the rumen that produce CAZymes consist of *Prevotella*, *Clostridium*, *Alistipes*, and *Eubacterium*, according to Shen et al. (2020) research. In the current study, it was found that there was a significant increase in the abundance of certain microbiota in the MG group. This led to changes in the internal environment of the rumen, which subsequently affected the function of the microbial population. As a result, the digestion of the ration was also impacted.

Metabolomics data can offer greater insight into the impact of diet on the rumen (Vinayavekhin et al., 2010). Notably, the differential metabolites screened in LG compared to WG were highly expressed in the WG group. Phosphatidylcholine [PC(16:0/0:0), PC(P-16:0/2:0), PC(18:3/0:0)] is a crucial component of cell membranes, playing a vital role in lipid metabolism and cellular activity. Phospholipidation is also essential for transporting substances into the cell (Santos and Lima, 2007). Shahsavari et al. (2016) discovered that choline plays an important role in preventing fatty liver and ketosis in high-yielding dairy cows, choline does this by contributing to sugar-lipid metabolism in the rumen. This study found that choline is highly expressed in both MG and HG, indicating that phosphatidylcholine is essential for assimilating high-energy diets. Intergroup differences were observed for 1,3,7-Trimethyluric acid, which is involved in caffeine metabolism and is a derivative of uric acid. The compound promotes dopamine and norepinephrine secretion (Tassaneeyakul et al., 1994). An increase in dopamine affects growth hormone (GH) synthesis and secretion, which promotes animal growth (Terry and Craig, 1985). Therefore, the increase in 1,3,7-Trimethyluric Acid in the MG group facilitated the animals’ growth. Glycerophosphoethanolamine [PE(18:0/0:0) and PE(P-16:0/0:0)] is vital for the synthesis of glycosylphosphatidylinositol-anchored proteins (GPI-AP) and promotes quick development of animal cells; however, it must be obtained through diet (Kano-Sueoka et al., 2001; Patel and Witt, 2017). Our hypothesis is that ethanolamine phosphate may contribute to muscle growth in yaks. Uridine 3′-monophosphate and Uridine-5′-monophosphate are pyrimidine nucleotides utilized as raw materials for RNA synthesis. Uridine monophosphate can cross the blood-brain barrier and upon absorption, undergoes conversion to cytidine triphosphate. This compound then combines with diacyl glycerol to form phosphatidylcholine, which subsequently participates in the biological process of phosphatidylcholine (Cornford and Oldendorf, 1975; Cansev, 2006). Previous studies have shown a correlation between changes in metabolite concentrations and alterations in the abundance of rumen microbiota (Huang et al., 2021). We found that differential microbiota such as *g__NK4A214_group*, *Papillibacter*, *norank*_*f__Muribaculaceae*, and *Family_XIII_AD3011_group* were significantly positively correlated with the screened differential metabolites, and it was mainly concentrated in MG group. This result further emphasizes the significance of rumen microbiota in producing high-concentration metabolites. Increasing the energy level of the ration enhanced TVFA concentration and growth performance in yak rumen. However, it led to a decrease in microbial abundance and metabolites. According to Chen Y. et al. (2012), a prolonged diet of high grain rations leads to an increase in the abundance of *Streptococcus bovis* in the rumen. This results in higher production of lactic acid and a decrease in rumen pH. In the present study, it was found that rumen flora counts were generally lower in the yaks of the HG group. This may be because the experimental samples were collected at the end of the feeding period, which changed the environment suitable for microbial survival during prolonged feeding of high-energy diets, inhibiting rumen microbial activity and ultimately affecting the productivity and rumen health of yaks.

## 5 Conclusion

We synthesized and analyzed the effects of dietary energy levels on yak growth performance by combining yak body weight data, rumen fermentation parameters, 16S rRNA, Metagenomics, and metabolomics data. The results of the experiment indicate that high energy-level rations increase VFA production in yaks, leading to a significant increase in their body weight. However, this increase was observed alongside a decrease in the abundance of rumen microorganisms and weakened functions related to cellulose degradation (e.g., GHs, CEs). The diet for medium energy groups not only increased the relative abundance of ruminal microbes and metabolites but also improved the Microbial ability to degrade carbohydrates. This diet was found to be effective in promoting weight gain while ensuring good rumen health. Therefore, it is recommended to use the medium energy group diet for fattening yaks during the Dry grass stage. Comprehensively analyzing the impact of energy level in rations on yak feeding basic data are provided for the development of the yak industry on the Tibetan Plateau. Furthermore, it delivers a new understanding regarding the metabolites and microbial functions of yaks.

## Data availability statement

The datasets presented in this study can be found in online repositories. The names of the repository/repositories and accession number(s) can be found below: Bioproject accession number PRJNA1017979.

## Ethics statement

The animal experiments involved in this experiment were approved by the Lanzhou Institute of Husbandry and Pharmaceutical Sciences of the Chinese Academy of Agricultural Sciences (CAAS) (approval number: 1610322020018). All sampling procedures are strictly in accordance with the Guidelines for Ethical Treatment of Experimental Animals in China. The studies were conducted in accordance with the local legislation and institutional requirements. Written informed consent was obtained from the owners for the participation of their animals in this study.

## Author contributions

XM: Data curation, Formal analysis, Methodology, Software, Visualization, Writing—original draft. YL: Validation, Writing—review and editing. GY: Supervision, Resources, Writing—review and editing. RD: Data curation, Writing—review and editing. JZ: Investigation, Writing—review and editing. YZ: Supervision, Writing—review and editing. JJ: Software, Writing—review and editing. XM: Data curation, Writing—review and editing. XG: Formal analysis, Writing—review and editing. MC: Formal analysis, Writing—review and editing. PY: Formal analysis, Project administration, Writing—review and editing. QZ: Formal analysis, Project administration, Supervision, Writing—review and editing. CL: Formal analysis, Funding acquisition, Project administration, Supervision, Writing—review and editing.

## Footnotes

1
https://ccb.jhu.edu/software/FLASH/index.shtml

2
https://github.com/OpenGene/fastp

3
http://www.drive5.com/usearch/

4
https://sourceforge.net/projects/rdpclassifier

5
https://www.mothur.org/wiki/

6
http://asia.ensembl.org/Bos_grunniens/Info/Index

7
http://bio-bwa.sourceforge.net

8
https://github.com/voutcn/megahit

9
http://www.cazy.org

10
http://www.genome.jp/kegg/

11
www.hmdb.ca

## Supplementary material

Supplementary Material 1 Classification status of differential metabolites.
