# Supplementary material for: Multi-omics revealed the effects of dietary energy levels on the rumen microbiota and metabolites in yaks under house-feeding conditions
Source: Front Microbiol. 2024 Jan 9;14:1309535. doi: 10.3389/fmicb.2023.1309535 (PMC10803511; doi:10.3389/fmicb.2023.1309535)
Supplement: Supplementary Material 1 — Classification status of differential metabolites. [file Data_Sheet_1.pdf]

Supplementary Material

1 Supplementary Figure 1

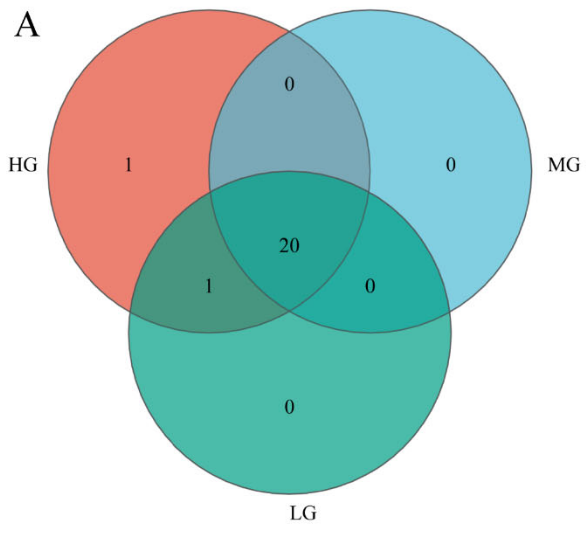

Supplementary Figure 1: The number of species shared and unique to the three treatment groups was counted using Venn diagrams. The three treatment groups overlap at the phylum (A) and genus (B) levels.

2 Supplementary Material S1

groups	Metabolite	M/Z
HG vs MG	Lipids and lipid-like molecules	
	Strictosidine	563.27
	(3beta,17alpha,23S)-17,23-Epoxy-3,29-dihydroxy-27-norlanosta-7,9(11)-diene-15,24-dione	471.31
	(1R*,2R*,4R*,8S*)-p-Menthane-1,2,8,9-tetrol 9-glucoside	408.22
	3-carboxy-4-methyl-5-pentyl-2-furanpropanoic acid	291.12
	2-Octenedioic acid	190.11
	Nucleosides, nucleotides, and analogues	
	Deoxyguanylic Acid	348.07
	Adenosine monophosphate	348.07
	DTMP	321.04
	Guanosine monophosphate	362.05
	Organic acids and derivatives	
	N2-Acetylornithine	139.09
	Maleic Acid	115.00
	Organic oxygen compounds	
	N-Acetyl-a-neuraminic acid	346.06
	N-Acetyl-D-Glucosamine 6-Phosphate	346.06
	Benzenoids	
	(4-Ethoxyphenyl)urea	181.10
HG vs LG	Lipids and lipid-like molecules	
	3,14-Dihydroxy-11,13-dihydrocostunolide	289.14
	4-Hydroxyproline galactoside	276.11
	5-Phenyl-1-pentanol	147.12
	Cis-12-Oxophytodienoic acid	257.19
	25-Hydroxyvitamin D3-26,23-lactol	453.30
	LysoPC(18:1(9Z)/0:0)	522.36
	LysoPC(18:1(11Z)/0:0)	544.34
	DG(20:0/0:0/18:2n6)	701.55
	LysoPE(20:1(11Z)/0:0)	530.32
	LysoPE(18:2(9Z,12Z)/0:0)	460.28
	PC(18:3/0:0)	518.32
	Jasmonic acid	193.12
	Oleyl alcohol	286.31
	PC(16:0/0:0)	496.34
	Ethyltestosterone	349.27
	LysoSM(d18:0)	484.38
	3-Hydroxy-beta-ionone	209.15
	KAPA	188.13
	Pentadecanoylcarnitine	422.27
	3,4-Methyleneadipic acid	215.06
	Organoheterocyclic compounds	
	Rockogenin	455.32

	2-Furanmethanol	99.04
	Xanthine	153.04
	Paraldehyde	196.09
	Adenine	134.05
	Oxypurinol	151.03
	Molybdopterin precursor Z	344.04
	5-Butyltetrahydro-2-oxo-3-furancarboxylic acid	231.09
	5-(3E-Pentenyl)tetrahydro-2-oxo-3-furancarboxylic acid	179.07
	1,3,7-Trimethyluric Acid	245.04
	Organic acids and derivatives	
	Monoethyl malonic acid	115.04
	Prolyl-Glutamine	285.16
	Glycylvaline	175.11
	Phenylacetylglycine	194.08
	Ecgonine Methyl Ester	200.13
	(2R,3R,4R)-2-Amino-4-hydroxy-3-methylpentanoic acid	148.10
	Glycyl-leucine	187.11
	Kainic acid	194.08
	Organic oxygen compounds	
	2-Hydroxyacorenone	259.17
	1-Deoxy-D-xylulose	176.09
	2,5-Dihydroxybenzaldehyde	137.02
	Glucosyl (E)-2,6-Dimethyl-2,5-heptadienoate	315.14
	Benzenoids	
	3,8-Dihydroxy-1-methylanthraquinone-2-carboxylic acid	340.08
	2,4,6-Trihydroxybenzoic acid	169.01
	2,4,6-Trihydroxybenzoic acid	109.02
	Phenylpropanoids and polyketides	
	3,8-Dihydroxy-9-methoxycoumestan	340.08
	Ellagic Acid	300.99
	Alkaloids and derivatives	
	Pseudoecgonine	218.14
	Organic nitrogen compounds	
	2-Methylpropanamine	74.10
	Homogeneous non-metal compounds	
	Pyrophosphate	176.94
MG vs LG	Organic acids and derivatives	
	Glutaminylalanine	218.11
	Glycyl-L-Tyrosine	239.10
	N-Methyl-L-Proline	147.11
	Methionine Sulfoxide	166.05
	L-2-Aminoadipic acid	162.08
	N-Acetylputrescine	131.12
	N-Acetyl-L-glutamate 5-semialdehyde	174.08
	5-Aminopentanoic acid	118.09
	Galactosyl 4-hydroxyproline	316.10
	Propionylglycine	132.07

Perindoprilat	323.20
N6-Acetyl-L-lysine	230.15
Glycylvaline	175.11
L-Hypoglycin A	159.11
Cyclo(proline-leucine)	228.17
Tyrosyl-Proline	279.13
Serylleucine	219.13
Epsilon-(Carboxyethyl)lysine	201.12
L-Aspartyl-L-Phenylalanine	281.11
Glutamylleucine	261.14
L-Ornithine	133.10
Islanditoxin	604.19
N-Acetyl-DL-Phenylalanine	208.10
N-Phenylacetylaspartic acid	252.09
L-phenylalanyl-L-proline	263.14
Ala-phe	237.12
Beta-Leucine	173.13
Penmacric acid	167.05
Gly-Ile	189.12
2-Aminoheptanedioic Acid	158.08
Gamma-L-Glutamyl-L-pipecolic acid	223.11
Pyroglutamic Acid	130.05
ALA-ILE	203.14
N-Methylvaline	132.10
Norleucine	132.10
D-alpha-Aminobutyric acid	86.06
L-Proline	157.10
Sarcosine	131.08
N2-Succinyl-L-ornithine	233.11
Alanylcysteine	237.03
L-Alanine	90.06
Glycylproline	190.12
D-Ala-D-Ala	161.09
L-Canaline	176.10
N-Carboxyethyl-g-aminobutyric acid	217.12
DL-Dopa	198.08
Allysine	187.11
Alpha-Aminobutyric acid	86.06
Isonicotinic acid	124.04
L-Glutamate	148.06
4-Amino-2-methylenebutanoic acid	160.06
Ureidoisobutyric acid	145.06
L-4-Hydroxyglutamate semialdehyde	168.03
D-Ornithine	131.08
DL-Methionine Sulfone	180.03
1-Aminocyclopropanecarboxylic Acid	100.04
L-Homocitrulline	188.10

L-Tyrosine	180.07
N-ACETYLPROLINE	156.07
Glycyl-leucine	187.11
N-Acetyl-DL-Valine	158.08
Gamma-Glutamylcysteine	295.06
3-Hydroxyglutaric Acid	147.03
L-3-Aminodihydro-2(3H)-furanone	146.05
N-Acetylleucine	172.10
D-(+)-Malic acid	133.01
Glutamylphenylalanine	275.10
Gamma-Glutamylphenylalanine	293.11
Leucyl-Glutamate	259.13
Gamma-Glu-leu	259.13
(S)-2-Azetidinecarboxylic acid	302.14
L-Alanyl-l-Valine	187.11
Gamma-Glutamylproline	289.10
Glutamylhydroxyproline	259.09
Asparaginyl-Valine	230.11
Glutamylglutamic acid	257.08
Beta-Alanine	88.04
Serylhydroxyproline	217.08
N-Acetyl-L-Glutamic Acid	188.06
N-alpha-Acetyl-L-citrulline	216.10
N-Acetylornithine	173.09
Succinic Acid	117.02
(+/-)-4-Methylene-2-pyrrolidinecarboxylic acid	172.06
Glutamylalanine	217.08
Glu-Gln	274.10
Citrulline	174.09
Hydroxyvalerylglycine	220.08
N-Methyl-L-Glutamic Acid	160.06
Methylmalonic Acid	117.02
L-Glutamic Acid	146.05
Malic Acid	133.01
Lipids and lipid-like molecules	
Senecioic acid	118.09
3,14-Dihydroxy-11,13-dihydrocostunolide	289.14
GPEtn(20:3/14:0)	714.51
4-Hydroxyproline galactoside	276.11
Malonylcarnitine	286.07
Pregnanetriolone	383.28
7a-Hydroxy-5b-cholanic acid	409.33
Docosapentaenoic acid (22n-3)	394.27
1-hexadecyl-glycero-3-phosphate	438.30
LysoPE(18:0/0:0)	504.31
LysoPC(18:1(9Z)/0:0)	522.36
LysoPC(18:1(11Z)/0:0)	544.34
Neotigogenin	439.32

LysoPE(20:2(11Z,14Z)/0:0)	488.31
LysoPE(0:0/18:0)	482.32
LysoPE(18:2(9Z,12Z)/0:0)	460.28
Strictosidine	563.27
CPA(16:0/0:0)	410.27
PC(18:3/0:0)	518.32
Withaperuvin H	596.29
Suspensolide F	511.23
(9Z,11E,13E,15Z)-4-Oxo-9,11,13,15-octadecatetraenoic acid	308.22
4-Hydroxyretinoic acid	334.24
2-Ethoxy-1-methyl-4-(1-methylethyl)benzene	220.17
3-carboxy-4-methyl-5-pentyl-2-furanpropanoic acid	291.12
2-Octenedioic acid	190.11
Prostaglandin D1	337.24
LysoPE(0:0/18:2(9Z,12Z))	478.29
PC(16:0/0:0)	496.34
LysoPE(16:0/0:0)	454.29
LysoPE(18:3(9Z,12Z,15Z)/0:0)	476.28
Tanacetol A	295.19
KAPA	188.13
9,10-Epoxyoctadecanoic acid	356.28
2-Isopropylmalic Acid	175.06
ACEXAMIC ACID	172.10
Gibberellin A126	391.14
(+)-15,16-Dihydroxyoctadecanoic acid	315.25
9-Hydroxy-10,12-Octadecadienoic Acid	295.23
(R)-3-Hydroxy-Octadecanoic acid	299.26
Tetradecanoylcarnitine	408.25
Cerebronic acid	383.35
LysoPE(0:0/16:0)	452.28
LysoPA(16:0/0:0)	455.24
2(R)-hydroxydocosanoic acid	355.32
PE(16:0/0:0)	452.28
Pentadecanoylcarnitine	422.27
Barogenin	429.30
12-hydroxyheptadecanoic acid	331.25
Secologanate	409.09
Gibberellin A29-catabolite	391.14
3,4-Methyleneadipic acid	215.06
Organoheterocyclic compounds	
2-Pyrrolidinone	86.06
Morpholino	70.07
1-(Hydroxymethyl)-5,5-dimethyl-2,4-imidazolidinedione	159.08
Delta-Valerolactam	100.08
5-Hydroxy-L-tryptophan	284.10
Sparstolonin B	286.07
Hypoxanthine	137.05

ortho-hydroxyatorvastatin	597.23
Melatonin	233.13
2-Isopropyl-6-Methylpyrimidin-4-ol	153.10
Mevalonolactone	95.05
4-Methyl-5-Thiazoleethanol	144.05
Guanine	152.06
Pyridoxamine	151.09
2-Hydroxyadenine	152.06
Xanthine	153.04
Pyrrolidine	72.08
1-Pyrroline	70.07
Paraldehyde	196.09
Alpha-[3-[(Hydroxymethyl)nitrosoamino]propyl]-3-pyridinemethanol	258.14
Uracil	113.03
Indole	118.07
Oxypurinol	151.03
Molybdopterin precursor Z	344.04
Lipoamide	186.04
P-Hydroxynordiazepam	285.04
7-Aminoclonazepam	320.03
Isoeugenitol	251.06
1,3,7-Trimethyluric Acid	245.04
Organic oxygen compounds	
Trans-2-pentenol	104.11
Beta-tyvelose	190.11
6-[5-(carboxymethyl)-2-hydroxyphenoxy]-3,4,5-trihydroxyoxane-2-carboxylic acid	309.06
4-Oxo-4-(3-pyridyl)-butanamide	161.07
6-[4-(carboxymethyl)-2-hydroxyphenoxy]-3,4,5-trihydroxyoxane-2-carboxylic acid	309.06
Viburtinal	178.09
2-Hexylidenecyclopentanone	205.10
2-Hydroxyacorenone	259.17
4-Hydroxybenzaldehyde	123.04
Sucrose	365.11
Mannan	689.21
Glyceric Acid	105.02
N-Acetyl-a-neuraminic acid	346.06
Stachyose	711.22
Glyceraldehyde	179.06
L-Ribulose	149.05
Glucosyl (E)-2,6-Dimethyl-2,5-heptadienoate	315.15
N-Acetyl-D-Glucosamine 6-Phosphate	346.06
Gluconolactone	177.04
Melezitose	503.16
D-Ribulose	195.05
Mannitol 1-phosphate	243.03

Threonic Acid	135.03
Maltotriose	539.14
N-Acetyl-D-galactosamine	266.09
Nucleosides, nucleotides, and analogues	
Nebularine	527.16
Arabinosylhypoxanthine	307.04
Allopurinol riboside	291.07
Guanosine	284.10
N6-Methyladenosine	282.12
Deoxyguanylic Acid	348.07
Adenosine monophosphate	348.07
Indole-3-Carboxaldehyde	146.06
CAMP	328.05
DTMP	321.05
Inosine	267.07
Xanthosine	283.07
Deoxyinosine	251.08
ADP-ribose	558.07
5-Thymidylic acid	321.05
Guanosine monophosphate	362.05
Phenylpropanoids and polyketides	
(E)-3-(2-Hydroxyphenyl)-2-propenal	166.09
Oxynarcotine	414.15
P-coumaroyltriacetic acid lactone	272.07
3,8-Dihydroxy-9-methoxycoumestan	340.08
S-6-Hydroxywarfarin	325.11
Trans-Resveratrol 3-O-glucuronide	369.10
Cis-Resveratrol 3-O-glucuronide	369.10
R-10-Hydroxywarfarin	325.11
2-Hydroxycinnamic acid	182.08
P-Coumaric Acid	182.08
P-Coumaroyl glycolic acid	267.05
Vinyl caffeate	251.06
Ellagic Acid	301.00
Alpha-Cyano-4-hydroxycinnamic acid	188.04
Organic nitrogen compounds	
Histidinal	140.08
(+/-)-2-(2-Furanyl)pyrrolidine	275.18
Phenylethanolamine	120.08
METHACHOLINE	160.13
Stearoylethanolamide	328.32
Ritalinic acid	264.12
P-Benzoquinone imine	152.03
Benzenoids	
4-Anilino-4-oxobutanoic acid	235.11
(4-Ethoxyphenyl)urea	181.10
Usnic acid	367.08

2,4,6-Trihydroxybenzoic acid	169.01
4-Methoxyphenylacetic acid	211.06
Homovanillin	211.06
Alkaloids and derivatives	
Arecaidine	174.11
1,2,3,4-Tetrahydro-b-carboline-1,3-dicarboxylic acid	305.08
Homogeneous non-metal compounds	
Pyrophosphate	176.94
